# Target-label-free artificial intelligence framework for cross-anatomical RNFL biomarker segmentation in optical coherence tomography

**DOI:** 10.3389/fcell.2026.1890734

**Published:** 2026-08-11

**Authors:** Suo Qiu, Juntao Zhang, Na Zhao, Xinxin Hu, Hengqian He, Leilei Yuan, Yaxuan Zhao, Shaodong Ma, Yitian Zhao, Qinkang Lu

**Affiliations:** 1 The Affiliated People’s Hospital of Ningbo University, The Eye Hospital of Wenzhou Medical University (Ningbo Branch), Ningbo, China; 2 Ningbo Key Laboratory for Neuroretinopathy Medical Research, Ningbo, China; 3 Laboratory of Advanced Theranostic Materials and Technology, Ningbo Institute of Materials Technology and Engineering, Chinese Academy of Sciences, Ningbo, China

**Keywords:** artificial intelligence applications, chronic diseases, imaging biomarkers, ocular diseases, optical coherence tomography, unsupervised domain adaptation

## Abstract

**Background:**

Effective glaucoma management relies on accurate optical coherence tomography (OCT) quantification of the circumpapillary retinal nerve fiber layer (cpRNFL) to track disease progression. However, the clinical translation of artificial intelligence (AI)-driven structural assessment is bottlenecked by scarce optic nerve head (ONH) annotations and coupled anatomical-hardware domain shifts.

**Methods:**

We propose Macula2Disc (M2D), a target-label-free, transductive AI framework for cross-anatomical image analysis. Trained exclusively on source-domain macular B-scans, M2D bridges the macula-to-disc gap across heterogeneous OCT platforms (Heidelberg Spectralis and TowardPi BMizar-400K) via three integrated components: (1) a Three-Level Deformation Module (TLDM) synthesizing ONH morphological variations from warp-stable macular topologies; (2) a Non-Uniform Rational B-Spline (NURBS) photometric module calibrating device-specific intensity manifolds; and (3) a Cross-Domain Segmentation Network (CDSN) optimized through entropy-gated global feature alignment.

**Results:**

Validation on 1,017 unannotated ONH circumpapillary scans (422 participants) yielded an overall RNFL Dice coefficient of 87.42% and an MIoU of 80.43%. Evaluated against expert consensus in a mixed-device clinical subset, M2D demonstrated statistical concordance (Mean Absolute Difference [MAD] = 1.8 μ m, ICC(2,1) = 0.96, P=0.62 ). In cases of pronounced RNFL attenuation, an advanced pathological state where proprietary commercial software exhibited statistically significant boundary-tracking deviations (MAD = 2.8 μ m, P=0.04 ), the proposed framework achieved anatomical alignment consistent with expert consensus. Across the entire cohort, M2D structural biomarker measurements demonstrated statistical agreement with commercial baseline outputs (Overall MAE = 1.5 μ m), indicating a robust technical capacity to approximate proprietary segmentations across different hardware architectures.

**Conclusion:**

The M2D framework provides a target-label-free AI strategy to resolve coupled macula-to-disc shifts. By approximating commercial anatomical measurements in general populations while resolving boundary-tracking vulnerabilities in pathological extremes, this approach establishes a foundational computational basis for cross-platform anatomical assessment. The primary endpoint of this study is the consistent technical evaluation of structural biomarker extraction, with rigorous diagnostic performance analysis remaining a crucial future direction.

## 1 Introduction

Glaucoma is a leading global cause of irreversible blindness and a quintessential chronic ocular disease. It is pathophysiologically characterized by the progressive degeneration of retinal ganglion cells (RGCs) and their axonal projections. Affecting over 76 million individuals worldwide, its prevalence is projected to escalate in the coming decades, primarily driven by demographic aging (Tham et al., 2014). A major clinical challenge in managing this chronic condition is that over 60% of individuals remain undiagnosed until moderate-to-late disease stages, after irreversible structural and functional decline has occurred (Allison et al., 2020). This delayed diagnosis is particularly evident in under-resourced primary care settings, where the absence of accessible, objective screening modalities exacerbates the socioeconomic burden on healthcare systems, highlighting an urgent need for adaptable, artificial intelligence (AI)-driven assessment technologies.

Optical coherence tomography (OCT) has emerged as a fundamental non-invasive imaging modality, significantly advancing the *in vivo* structural assessment of ocular neuroanatomy. Longitudinal cohort studies indicate that OCT can identify retinal nerve fiber layer (RNFL) thinning 3–6 years prior to the manifestation of visual field defects on standard automated perimetry (Hood et al., 2022). Specifically, the circumpapillary RNFL thickness (cpRNFLT) decreases by an estimated average of 2.3 μ m per year in early-stage glaucoma, preceding overt functional impairment (Leung et al., 2013). Consequently, cpRNFL quantification provides a sensitive structural biomarker for early glaucomatous damage. However, translating this biomarker into large-scale AI applications for consistent anatomical evaluation necessitates robust, automated extraction methods capable of operating across diverse clinical environments (Devalla et al., 2020; Thompson et al., 2020b; Yang et al., 2025).

**FIGURE 1:**
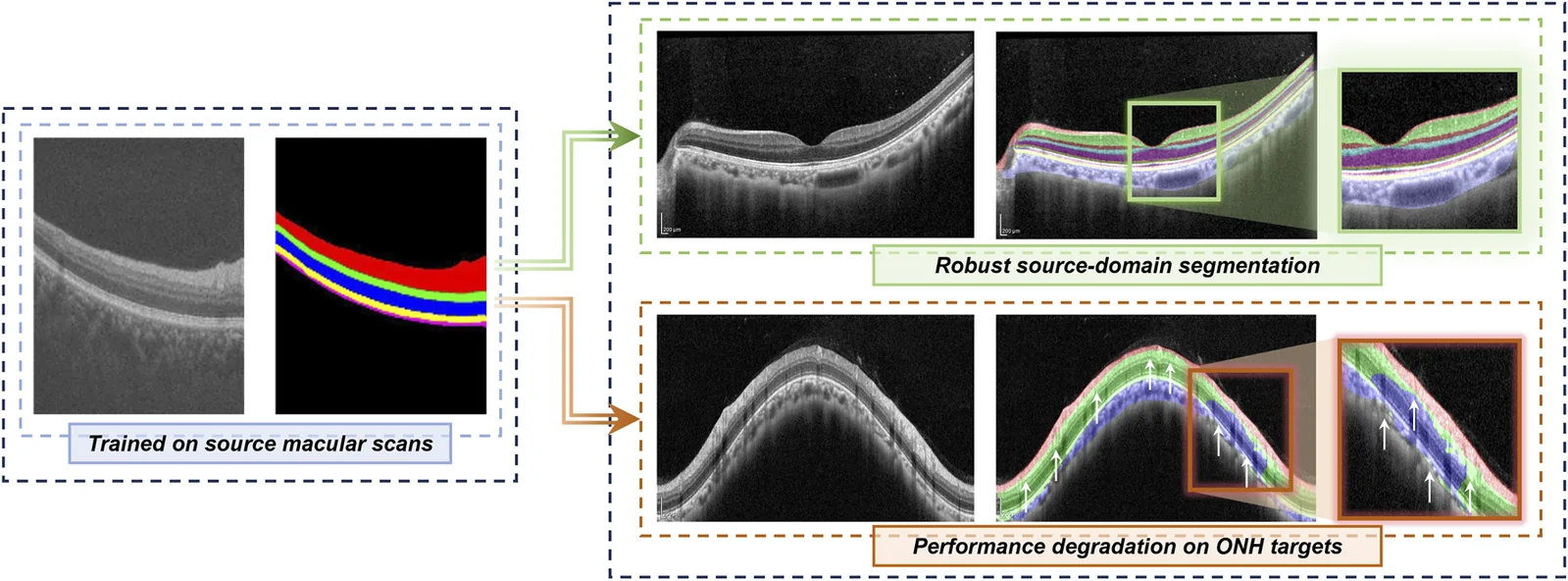
Visualization of the cross-anatomical domain shift and resultant segmentation failure. The Macular-trained Baseline U-Net (MB-U-Net), trained exclusively on macular B-scans, yields stable source-domain delineations (top right). However, direct inference on target-domain ONH circumpapillary scans (bottom right) induces pronounced boundary-tracking deviations, notably within the RNFL. White arrows indicate localized structural misalignments.

Despite the growing utility of AI in ophthalmology, the widespread clinical deployment of automated RNFL assessment is frequently impeded by coupled anatomical and hardware-induced domain shifts (Guan and Liu, 2021). In the context of artificial intelligence, a “domain shift” refers to a significant change in the underlying data distribution between the training and testing environments that typically degrades algorithmic performance. In this study, this shift is explicitly twofold: an *anatomical* domain shift (the spatial transition of the imaging target from the macular region to the optic nerve head [ONH]) and a *hardware-induced* domain shift (the transition of image acquisition across disparate OCT manufacturers). Currently, many state-of-the-art OCT segmentation models are trained on macular B-scans, utilizing morphologically stable retinal layers and the abundance of annotated public datasets (Fang et al., 2017; Wu et al., 2023). However, applying these predictive models directly to ONH circumpapillary scans often exposes critical generalization limitations. Although the ONH is a primary site for clinically actionable RNFL evaluation, macular-trained algorithms frequently experience a decline in segmentation fidelity upon this anatomical transition (Figure 1). This performance degradation is critically exacerbated in realistic cross-device scenarios. For instance, a model trained exclusively on source-domain macular scans from one platform (e.g., Optovue RTVue XR) faces compounded domain shifts when applied to target-domain ONH scans acquired by disparate optical systems (e.g., Heidelberg Spectralis or TowardPi BMizar-400K). This coupled cross-anatomical and cross-device domain shift, driven by structural disparities (such as optic cup geometry) alongside hardware-specific photometric variations, presents a substantial technical barrier for the robust deployment of AI models across diverse imaging platforms (Lu et al., 2025).

Furthermore, the manual delineation of highly attenuated retinal layers in ONH circumpapillary scans remains a labor-intensive, inherently subjective task requiring specialized clinical expertise. This scarcity of pixel-level annotations restricts the supervised training of robust AI architectures (Tajbakhsh et al., 2020). This limitation motivates the development of an AI framework capable of leveraging abundant macular priors to reliably extract RNFL biomarkers from unannotated ONH targets, thereby broadening accessibility across closed-source commercial hardware. We explicitly clarify that the primary endpoint of this study is the reliable extraction of structural biomarkers, rather than direct diagnostic classification.

To bridge this translational gap, we propose Macula2Disc (M2D), an AI framework for cross-anatomical RNFL biomarker segmentation via unsupervised domain adaptation. Our primary contributions are as follows:

- We introduce the M2D framework, an AI architecture integrating three core components: a topology-preserving Three-Level Deformation Module (TLDM), a Non-Uniform Rational B-Spline (NURBS) photometric module, and a Cross-Domain Segmentation Network (CDSN). This approach effectively addresses coupled macula-to-disc domain shifts, enabling circumpapillary biomarker extraction driven exclusively by macular structural priors.
- We demonstrate the cross-platform consistency of this framework across a heterogeneous clinical cohort of 1,017 independent ONH scans. By approximating the baseline outputs of distinct OCT platforms (Heidelberg Spectralis and TowardPi BMizar-400K), M2D provides a consistent computational approach for cross-device structural assessment.
- We demonstrate the framework’s application in extracting biomarkers under advanced pathological states characterized by pronounced RNFL attenuation. By mitigating the boundary-tracking limitations observed in proprietary commercial algorithms during advanced disease stages, the model yields anatomical measurements consistent with adjudicated expert consensus, directly providing a reliable structural baseline for cross-platform anatomical evaluation.

## 2 Materials and methods

### 2.1 Study design overview

This cross-sectional study followed a three-stage workflow: (1) *AI Baseline Evaluation and Domain Gap Characterization* via a macular-trained baseline network; (2) *Framework Development* of the M2D dual-space augmentation pipeline and CDSN architecture; and (3) *Comprehensive Clinical Evaluation* assessing cross-device consistency on 1,017 ONH circumpapillary scans (Heidelberg and TowardPi), clinical concordance within a 100-scan mixed cohort, and structural stratification across a 422-participant evaluation cohort. Retrospective data collection between June 2024 and September 2025 adhered to the Declaration of Helsinki and was approved by the Ethics Committee of The Affiliated People’s Hospital of Ningbo University, China (Approval No. 2024-067).

To rigorously prevent data leakage, all dataset partitions were executed strictly at the patient-level. For the source-domain macular cohort, we eliminated intra-subject correlation by including a single randomly selected volume per patient (n=105) . Uniform spatial sampling of 30 B-scans per $6\times 6\;{\text{mm}}^{2}$ volume (Optovue RTVue XR) mitigated adjacent-slice redundancy, yielding 3,150 curated B-scans. This corpus was partitioned via a standard 80:20 patient-level split into training (84 patients, 2,520 scans) and validation (21 patients, 630 scans) sets. A standard U-Net (Ronneberger et al., 2015) (MB-U-Net) was trained exclusively on this source data to quantify the baseline domain shift. Crucially, the target ONH cohort—comprising 422 participants (1,017 circumpapillary scans; one optimal scan per eye per modality)—was utilized exclusively as unannotated data during the global alignment training phase and subsequently evaluated for segmentation performance. This transductive unsupervised domain adaptation setting accurately reflects realistic cross-device deployment. Specifically, it simulates scenarios where unannotated target-domain scans are available for algorithm calibration prior to inference, thereby obviating the need for paired dual-scan acquisitions.

While achieving a source-domain segmentation Dice coefficient of 83.45%, the MB-U-Net exhibited an 18.3% performance degradation when directly applied to external ONH scans. As visualized in Figure 1, this direct inference induces pronounced boundary-tracking deviations, particularly within the RNFL. This deficit highlights the non-transferability of source-domain priors (Guan and Liu, 2021), driven by coupled hardware and anatomical shifts:

1. *Photometric Variations*: Inter-device optical discrepancies and proprietary speckle-reduction algorithms yield heterogeneous intensity profiles (Ma et al., 2019).
2. *Topological Disparities*: Unlike the relatively planar macula, ONH scans inherently exhibit complex optic disc elevation, neuroretinal rim tapering, and asymmetric RNFL distributions.

Given that conventional affine augmentations inadequately address these non-linear discrepancies, the M2D framework was developed to explicitly synthesize structural and photometric variations, thereby mitigating this domain gap.

### 2.2 Dual-space data augmentation framework

**FIGURE 2:**
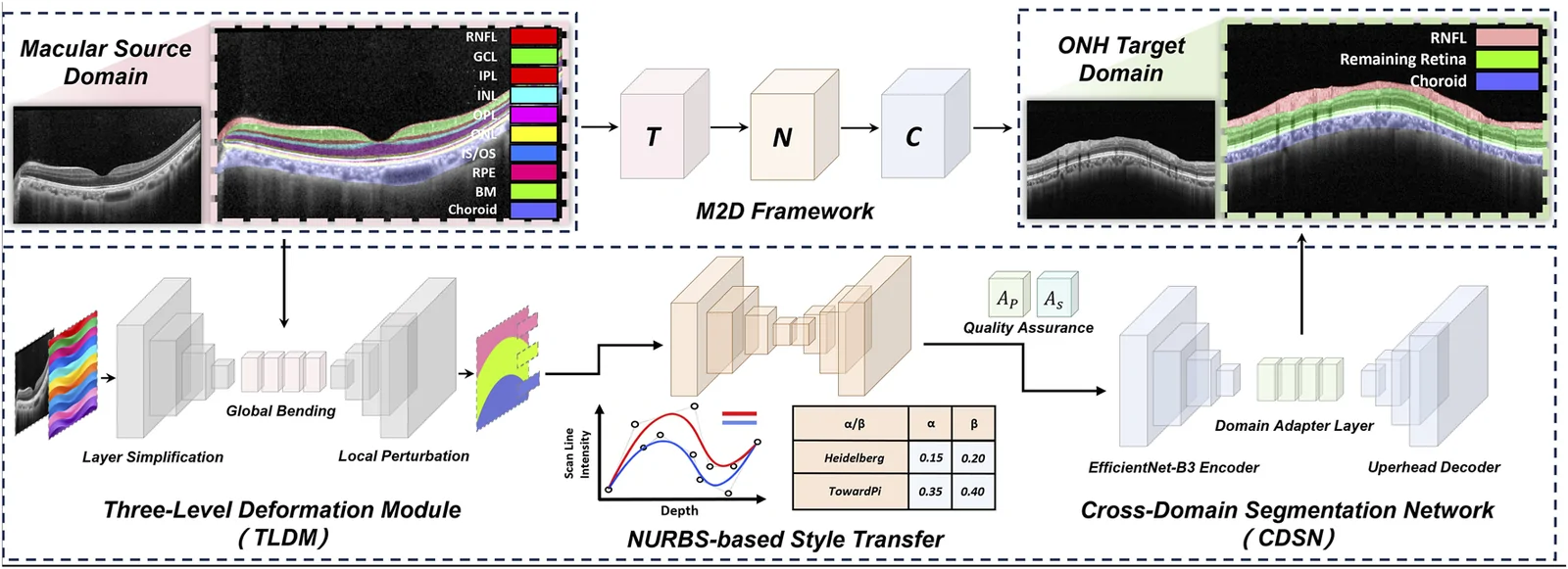
Architecture of the target-label-free Macula2Disc (M2D) framework. The pipeline synthesizes pseudo-ONH representations from macular B-scans via three components: (1) a Three-Level Deformation Module (TLDM) abstracting ten-layer annotations into a warp-stable three-layer topology for elastic perturbation; (2) a Non-Uniform Rational B-Spline (NURBS) module for device-specific photometric calibration; and (3) a Cross-Domain Segmentation Network (CDSN) featuring a Domain Adapter Layer. Synthetic pairs undergo topological quality assurance filtering prior to network optimization.

Drawing on single-source domain generalization principles (Wang et al., 2020; Lu et al., 2025), the complete M2D framework (illustrated in Figure 2) explicitly disentangles the domain shift into two orthogonal augmentation manifolds (spatial and appearance).

#### 2.2.1 Spatial Structure Space $\left(A_{s}\right)$ – Geometric warping via TLDM

To operationalize the spatial manifold $A_{s}$ , we developed the Three-Level Deformation Module (TLDM), which abstracts standard ten-layer macular annotations into a warp-stable, three-layer representation prior to applying dense elastic transformations. This mitigates non-physiological inter-layer intersections (slippage) under extensive geometric warping.

Layer-0 (RNFL) is maintained as the primary biomarker target. Layer-1 (Remaining Retina, RR) is formulated via a dynamically learned convex combination of existing anatomical boundaries:

$$y_{RR}\left(x\right)=\alpha_{s1}\cdot y_{GC}\left(x\right)+\alpha_{s2}\cdot y_{\mathrm{MRC}}\left(x\right)+\left(1-\alpha_{s1}-\alpha_{s2}\right)\cdot y_{\mathrm{ORB}}\left(x\right)$$ (1)

where x denotes the horizontal position (A-scan column) across the OCT image, and $y_{RR}\left(x\right)$ represents the newly computed vertical depth (spatial y -coordinate) for the consolidated RR pseudo-boundary. Correspondingly, $y_{GC}\left(x\right)$ , $y_{\mathrm{MRC}}\left(x\right)$ , and $y_{\mathrm{ORB}}\left(x\right)$ denote the original spatial y -coordinates of the Ganglion Cell Complex, Mid-Retina Composite, and Outer Retina Boundary at column x . This geometric interpolation physically collapses multiple intermediate retinal layers into a single, stable structural anchor. Fusion weights $\alpha_{s1},\alpha_{s2}\in \left[0.2,0.4\right]$ are optimized to maximize deformation stability. Layer-2 (Choroid Complex, CC) serves as a rigid inferior anchor.

Crucially, merely altering the pixel labels (the segmentation mask) without applying corresponding physical deformation to the underlying OCT image would cause the new anatomical labels to structurally contradict the native image features, such as reflectivity gradients and micro-vascular shadows. Thus, the TLDM’s dense elastic warping is mathematically and physiologically essential to ensure the network learns biologically consistent mapping rules. The TLDM applies synchronized transformations to approximate ONH topography: dense Thin-Plate Spline (TPS) warping (Bookstein, 2002) controlled by a curvature parameter $\kappa \in \left[-0.02,0.02\right]\;{\text{mm}}^{-1}$ for global bending, and a high-frequency sinusoidal function f(x)=A⁡sin(2πx/Λ) ( A∈[2,8] , Λ∈[100,200] ) to simulate local micro-vasculature shadowing artifacts.

#### 2.2.2 Pixel-level appearance space $\left(A_{p}\right)$ – NURBS-based photometric mapping

Calibrated via unsupervised Bayesian optimization to minimize the 1D Wasserstein distance between source and target intensity histograms, a NURBS-based module operating strictly within the photometric domain aligns device-specific intensity manifolds while preserving local texture gradients.

Although the transformation modulates a 1D pixel intensity value, the non-linear contrast mapping curve itself is defined by control points in a 2D Cartesian plane: $\left(X_{\text{input\_intensity}},Y_{\text{output\_intensity}}\right)$ . From unlabeled target scans, we extract the intensity range $V\in \left[V_{l},V_{h}\right]$ , contrast dispersion σ , and distribution skewness S to parameterize the critical control points $P_{1}$ and $P_{2}$ .

$$P_{1}=\left(V_{l},V_{l}+\alpha \cdot \sigma \cdot e^{-\gamma |S|}\right),$$ (2)

$$P_{2}=\left(V_{h}-\beta \cdot \sigma \cdot e^{-\gamma |S|},V_{h}\right),$$ (3)

where the first element of each point dictates the input source intensity, and the second element dictates the targeted output intensity. The damping factor γ=0.1 mitigates mapping distortions under extreme skewness. Device-specific coefficients (α,β) are detailed in Table 1.

**TABLE 1:** NURBS parameter calibration for device-specific intensity mapping.

Device/Model	α (post-adapt)	β (post-adapt)	Physical interpretation
Heidelberg Spectralis	0.12±0.02	0.18±0.03	Balanced contrast; low speckle noise
TowardPi BMizar-400K	0.38±0.04	0.42±0.05	Narrow dynamic range; suppressed signal

NURBS: Non-Uniform Rational B-Spline. Values shown are means ± standard deviation from three optimization runs.

#### 2.2.3 Topology-preserving quality assurance

During the training phase, stochastic samples are dynamically generated on-the-fly via the random parametric sampling of the TLDM and NURBS modules. This stochastic sampling is essential to simulate the wide, unpredictable, and naturally occurring variations in optic disc morphology observed across diverse clinical populations, thereby robustly expanding the training distribution. To ensure biological plausibility, these on-the-fly augmented samples are gated using a Clinically Plausible Structure Deviation (CPSD) metric:

$$\text{CPSD}=\theta_{1}I_{\mathrm{frac}}+\theta_{2}N_{\mathrm{self}}+\theta_{3}\Delta A_{\mathrm{RNFL}}$$ (4)

where $I_{\mathrm{frac}}$ , $N_{\mathrm{self}}$ , and $\Delta A_{\mathrm{RNFL}}$ represent normalized indices quantifying non-physiological RNFL fragmentation, boundary self-intersections, and relative cross-sectional area changes, respectively.

Specifically, $I_{\mathrm{frac}}$ is computed as the normalized count of disconnected mask components to evaluate structural continuity. $N_{\mathrm{self}}$ is defined as a binary penalty index indicating any occurrence of topological crossing between upper and lower layer boundaries. $\Delta A_{\mathrm{RNFL}}$ is calculated as the absolute relative change in cross-sectional pixel area before and after deformation: $|A_{\mathrm{post}}-A_{\mathrm{pre}}|/A_{\mathrm{pre}}$ .

Using empirically validated weights $\left(\theta_{1},\theta_{2},\theta_{3}\right)=\left(0.5,0.3,0.2\right)$ , any stochastically generated sample yielding CPSD>0.15 (an empirically determined threshold optimized via grid-search to balance anatomical diversity with biological plausibility) or violating strict anatomical layer ordering is automatically discarded. To preclude infinite loops during extreme edge-case generation, a maximum of five resampling attempts is permitted per image before defaulting to an identity transformation. This online synthesis strategy expands the training corpus to promote model convergence while circumventing offline storage constraints.

### 2.3 Cross-domain segmentation network (CDSN) and optimization

**FIGURE 3:**
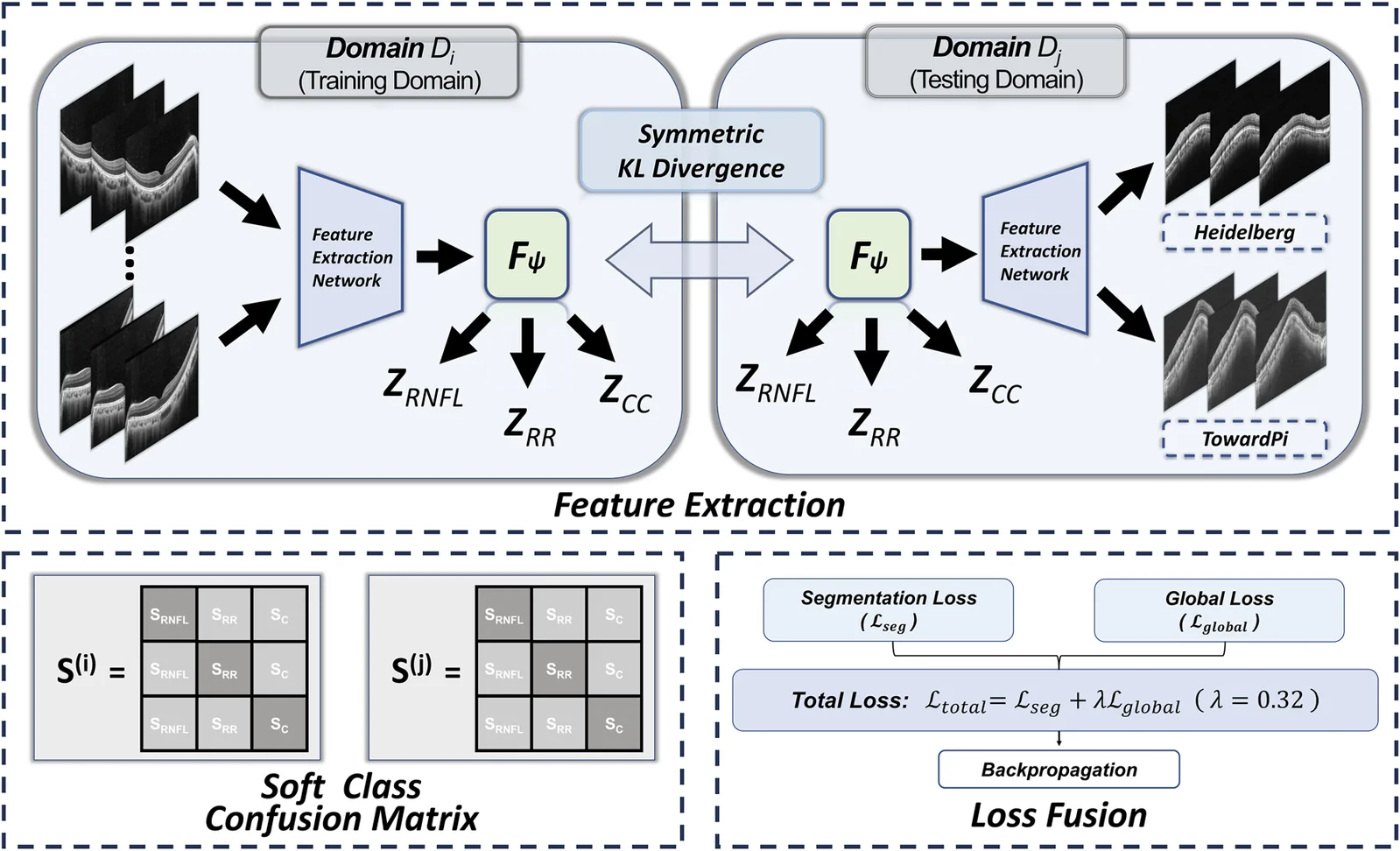
Computational pipeline for global class alignment via pseudo-prototypes. Solid lines represent the forward propagation: source-domain B-scans and unlabeled target-domain ONH circumpapillary scans traverse a shared feature encoder to yield soft prediction probabilities (S) . The symmetric Kullback-Leibler divergence quantifies the global alignment loss $\left(L_{\mathrm{global}}\right)$ . Dashed lines indicate the gradient flow, where $L_{\mathrm{global}}$ backpropagates through the Domain Adapter Layer (DAL) to establish feature-level domain alignment.

The CDSN employs an ImageNet-pretrained EfficientNet-B3 encoder (Tan and Le, 2019) paired with a UPerHead decoder (Xiao et al., 2018). A Domain Adapter Layer (DAL) is prepended to the final classifier, allowing backpropagation of the global alignment loss $\left(L_{\mathrm{global}}\right)$ to establish domain-invariant feature spaces. The computational pipeline for this domain-alignment process is detailed in Figure 3. The network is optimized using a dynamically weighted hybrid loss function:

$$L_{\mathrm{total}}=\left(L_{\mathrm{Dice}}+\mu L_{CE}\right)+\lambda \left(\text{epoch}\right)\cdot L_{\mathrm{global}}$$ (5)

where $L_{\mathrm{Dice}}$ represents the Dice similarity loss for global spatial overlap, $L_{CE}$ denotes the standard cross-entropy loss for local voxel-wise classification accuracy, and μ is an empirical weighting factor (set to 1.0) to balance these two supervised objectives.

#### 2.3.1 Target-label-free global alignment

To bypass target-domain annotations, $L_{\mathrm{global}}$ leverages a cross-domain aligned pseudo-prototype mechanism (Zou et al., 2019). Source-domain prototypes $z_{c}^{\left(s\right)}=\frac{1}{N_{c}^{\left(s\right)}}\sum_{n:yn\left(s\right)=c}F_{\psi }\left(x_{n}^{\left(s\right)}\right)$ seed the target-domain pseudo-prototypes $z_{c}^{\left(t\right)}$ via k -means clustering. To mitigate semantic drift, an Expectation-Maximization soft assignment is applied to each target-domain pixel-level feature vector $f_{t}^{\left(i\right)}$ :

$$p\left(c|f_{t}^{\left(i\right)}\right)=\frac{\exp\left(-\|f_{t}^{\left(i\right)}-z_{c}^{\left(t\right)}{\|}^{2}/T\right)}{\sum_{j=1}^{3}\exp\left(-\|f_{t}^{\left(i\right)}-z_{j}^{\left(t\right)}{\|}^{2}/T\right)}$$ (6)

where the temperature factor T=0.1 sharpens the assignment distribution across the three anatomical target classes.

Ambiguous pseudo-labels are suppressed via Shannon entropy-derived confidence weights $w_{c}$ . To ensure scale-invariance across varying resolutions, the unnormalized entropy is averaged over the $N_{t}$ target pixels:

$$w_{c}=\exp\left(\frac{\tau }{N_{t}}\sum {i=1}^{N_{t}}p\left(c|f_{t}^{\left(i\right)}\right)\log p\left(c|f_{t}^{\left(i\right)}\right)\right)$$ (7)

with the entropy decay factor set to τ=2.0 . Finally, global alignment minimizes the symmetric Kullback-Leibler (KL) divergence across active classes $\left(C^{\prime }\right)$ , gated by a strict confidence threshold ϵ=0.8 :

$$L_{\mathrm{global}}=\frac{1}{2C^{\prime }}\sum_{c:w_{c}\geq \epsilon }w_{c}\left[D_{KL}\left(S_{c}^{\left(s\right)}\|S_{c}^{\left(t\right)}\right)+D_{KL}\left(S_{c}^{\left(t\right)}\|S_{c}^{\left(s\right)}\right)\right]$$ (8)

where $S_{c}^{\left(s\right)}$ and $S_{c}^{\left(t\right)}$ denote the network’s spatial softmax probability distributions for class c in the source and target domains, respectively.

#### 2.3.2 Implementation details and curriculum training

The M2D framework was implemented in PyTorch v2.1 (Ubuntu 20.04 LTS) and trained on a single NVIDIA RTX 4090 GPU with a batch size of 16. During preprocessing, all OCT B-scans were resized to 512×512 pixels and normalized to a [0,1] intensity range. To ensure deterministic reproducibility and quantify algorithmic variance, global random seeds were strictly fixed; specifically, three independent experimental runs were executed using seeds 42, 123, and 456. To strictly preserve the target-label-free integrity of the framework, hyperparameters were tuned via grid search based on segmentation performance exclusively on the sequestered source-domain validation set. The hyperparameter search space encompassed base learning rates $\in \left[1\times 10^{-5},1\times 10^{-3}\right]$ , batch sizes ∈{8,16,32} , and the supervised loss weighting factor μ∈[0.5,2.0] . While unannotated target-domain (ONH) scans were fed into the network to compute the global alignment loss during Phase II, strictly no target-domain annotations were utilized during any phase of model training or hyperparameter optimization.

A two-phase curriculum was employed to mitigate catastrophic forgetting. The total training duration was empirically fixed at 500 epochs based on the convergence plateau observed on the source validation set. Phase I (Epochs 1–100) utilizes the supervised hybrid segmentation loss $\left(L_{\mathrm{Dice}}+\mu L_{CE}\right)$ . Phase II (Epochs 101–500) introduces the global alignment loss $\left(L_{\mathrm{global}}\right)$ via a 20-epoch linear warmup to ensure stability, followed by a constant weight plateau:

$$\lambda \left(\text{epoch}\right)=\left\{\begin{array}{cc} 0, & \text{epoch}\leq 100\\ 0.1+0.22\cdot \frac{\text{epoch}-100}{20}, & 100<\text{epoch}\leq 120\\ 0.32, & \text{epoch}>120 \end{array}\right.$$ (9)

Optimization was performed using Adam (weight decay = $1\times 10^{-4}$ ). Following the hyperparameter search, to preserve pre-trained ImageNet priors, layer-wise learning rates were applied: $1\times 10^{-4}$ for the EfficientNet-B3 encoder and $5\times 10^{-4}$ for the UPerHead decoder and Domain Adapter Layer. Gradient clipping (maximum norm = 5.0) was enforced to prevent gradient explosion during initial alignment. The model weights yielding the highest validation Dice coefficient were retained for final inference.

### 2.4 Statistical analysis

Statistical analyses were conducted to evaluate algorithmic performance, demographic distributions, and clinical concordance. Continuous variables were presented as mean ± standard deviation (SD). Spatial overlap was quantified using the Dice Similarity Coefficient (Dice) and mean Intersection over Union (MIoU). Measurement errors were assessed via Mean Absolute Difference (MAD) and Mean Absolute Error (MAE). To rigorously represent measurement uncertainty and algorithmic stability, point estimates for these principal performance metrics are reported alongside their 95% CIs. Furthermore, performance variance across repeated training runs with different random initializations is expressed as mean ± SD. Inter-method and inter-expert agreements were evaluated using the Intraclass Correlation Coefficient [ICC(2,1)], Lin’s Concordance Correlation Coefficient (CCC), and Bland-Altman Limits of Agreement (LOA).

Crucially, to account for intra-subject correlation (clustering) arising from the inclusion of bilateral eye scans from the same participants, Generalized Estimating Equations (GEE) with cluster-robust standard errors were strictly employed for all comparative statistical hypothesis testing (including demographic comparisons and measurement variance evaluations). This approach explicitly adjusts for within-patient variance, preventing the underestimation of standard errors inherent to simple independent tests. A predefined equivalence margin of ±5 μm was utilized to assess statistical measurement equivalence based on the 95% confidence interval (CI) of the mean bias. Clinically, this margin is robust as it corresponds to standard OCT axial optical resolution and aligns with the established test-retest variability (reproducibility) of circumpapillary RNFL measurements in standard clinical practice, which typically ranges from four to 6 μm (Mwanza et al., 2010). Any measurement difference within this threshold falls strictly within the inherent physiological noise floor and is considered clinically equivalent (Thompson et al., 2020a). To explicitly evaluate whether cross-platform consistency was disproportionately affected by clinical severity, a formal two-way interaction term (Device × Diagnosis) was also evaluated within the GEE framework. All statistical analyses were performed using R software version 4.2.2, utilizing the *geepack* package for GEE models. A two-sided P -value of <0.05 was considered statistically significant.

## 3 Results

To evaluate the technical viability of the proposed M2D AI framework, validations were conducted across three complementary streams: (1) cross-platform measurement agreement on 1,017 independent ONH circumpapillary scans from heterogeneous OCT platforms; (2) segmentation accuracy against adjudicated expert consensus within a 100-scan mixed-device subset; and (3) structural subgroup stratification across a 422-participant clinical cohort.

### 3.1 Evaluation metrics

To align with our dual validation objectives, reference standards were established cohort-specifically to ensure methodological rigor. The 1,017-scan evaluation cohort predominantly comprises healthy and mildly affected anatomies, a domain where commercial OCT algorithms operate reliably. Within this group, automated device outputs served as a *baseline comparator* to evaluate general anatomical concordance and cross-platform statistical consistency. By contrast, the 100-scan mixed-device subset was purposefully curated to capture advanced RNFL attenuation. Given that proprietary commercial software is susceptible to boundary-tracking failures in these severely thinned regions, automated outputs were deemed unsuitable as a reference. Therefore, the adjudicated expert consensus was utilized as the *ground truth* to rigorously demonstrate the robust pathological fidelity of the proposed framework. It is critical to state that the large-cohort analysis exclusively evaluates measurement agreement with commercial software, rather than accuracy against an independent ground truth. Because commercial software can exhibit boundary-tracking failures in advanced disease stages, all claims regarding the framework’s segmentation accuracy and pathological superiority are strictly based on the expert-annotated 100-scan subset.

Pixel-level spatial overlap was quantified using the Dice and MIoU:

$$\text{Dice}=\frac{2|X\cap Y|}{\left|X|+|Y\right|},\;\text{MIoU}=\frac{1}{C}\sum_{c=1}^{C}\frac{\left|X_{c}\cap Y_{c}\right|}{\left|X_{c}\cup Y_{c}\right|}$$ (10)

where X and Y denote the predicted and cohort-specific reference masks, respectively, across C=3 anatomical classes (RNFL, Remaining Retina [RR], and Choroid Complex [CC]).

### 3.2 Technical validation cohort and computational efficiency

The target-domain evaluation cohort exhibited substantial clinical and demographic heterogeneity, accurately reflecting a complex real-world clinical environment across 422 participants (yielding 1,017 total circumpapillary scans). As summarized in Table 4, the cohort encompassed a diverse clinical case mix: 108 normal controls, 102 cataract controls, 110 glaucoma suspects, and 102 confirmed glaucoma patients. Clinical parameters demonstrated profound variance corresponding to disease severity (P<0.001) . For instance, mean intraocular pressure (IOP) escalated from 14.2±2.9 mmHg in normal controls to 22.4±7.5 mmHg in confirmed cases, while the vertical cup-to-disc ratio (VCDR) spanned from healthy morphology (0.34±0.09) to advanced glaucomatous cupping (0.78±0.13) .

This specific cohort distribution and the resulting hardware-specific variance are direct consequences of our clinical data sampling methodology. To mirror realistic clinical acquisition pathways, 613 Heidelberg scans were sourced from systems deployed across both community clinic sites and the tertiary eye center, thereby capturing a broad demographic continuum from healthy populations to complex referrals. Conversely, the remaining 404 scans (comprising 202 sequentially paired cases) were acquired from a specialized glaucoma center where the TowardPi platform was exclusively stationed alongside a second Heidelberg unit. This deployment inherently captured a higher proportion of confirmed, advanced glaucoma cases on the TowardPi device. To ensure baseline reliability and mitigate signal-quality confounding across these varying demographics, all OCT scans underwent strict quality control in accordance with OSCAR-IB criteria and APOSTEL recommendations prior to inclusion. Specifically, a minimum image-quality score of >15 dB was mandated for Heidelberg scans, while a quality score of ≥7 was required for TowardPi scans.

This heterogeneous cohort reflects hardware-induced domain shifts arising from distinct acquisition protocols. Imaging was performed using the Spectralis OCT system (Heidelberg Engineering GmbH, Heidelberg, Germany), equipped with HEYEX two software (Version 6.16.11), which utilizes an 870-nm superluminescent diode operating at 40,000 A-scans per second. This system captures direct 3.45-mm diameter circular B-scans (768 A-scans per image), leveraging active eye-tracking and real-time image averaging to enhance signal quality. Conversely, swept-source OCT imaging was conducted using the BM-400K system (BMizar; TowardPi Medical Technology, Beijing, China). This platform employs a 1060-nm laser at 400,000 A-scans per second to acquire $6\times 6\;{\text{mm}}^{2}$ volumetric grids ( 512×512 pixels), from which a 3.4-mm circumpapillary calculation circle is computationally extracted.

Despite its algorithmic complexity, the M2D framework maintains practical computational efficiency. The model comprises approximately 26.8 million parameters (107.6 MB in size) and requires a peak inference memory footprint of 2.1 GB. Evaluated on a standard workstation (NVIDIA RTX 4090 GPU), the average inference latency is 37 m per scan. Furthermore, to assess computational feasibility for primary care settings lacking GPU infrastructure, benchmarking on a conventional CPU (Intel Core i7-12700K) yielded a processing time of approximately 418 m per scan. These baseline metrics indicate that the framework operates adequately on standard hardware, suggesting the potential for future integration into computational evaluation pipelines as an algorithmic adjunct.

#### 3.2.1 Quantitative agreement with commercial baselines

Compared with six established domain generalization methods under target-label-free conditions (Table 2, Panel A), the M2D framework improved composite three-layer segmentation agreement with the commercial baseline across target ONH domains. On the TowardPi device, characterized by a narrow dynamic range and distinct speckle noise, the model yielded a Dice agreement of 92.09% (95% CI: 90.63%–93.55%; mean ± SD across three random seeds: 91.24% ± 1.01%), an improvement of 3.83% over AugMix. This indicates the comparative advantage of NURBS-based photometric calibration over linear histogram matching in this setting.

**TABLE 2:** Quantitative cross-domain segmentation on target datasets.

Methods	Heidelberg (n=815)		TowardPi (n=202)	
	Dice (%)	MIoU (%)	Dice (%)	MIoU (%)
Panel A: Multi-Layer Joint Segmentation (3-Layer Average)				
MB-U-Net (Ronneberger et al., 2015)	84.04 (83.02–85.11)	74.02 (72.85–75.19)	88.62 (85.81–91.43)	80.66 (77.72–83.60)
SLAug (Su et al., 2023)	81.90	71.27	82.78	76.53
nnU-Net (Isensee et al., 2021)	82.35	75.72	81.78	77.42
RandAugment (Cubuk et al., 2020)	81.17	69.40	85.67	78.84
MedAugment (Liu e tal., 2023)	81.27	72.02	83.87	78.75
MixStyle (Zhou et al., 2021)	78.92	69.17	83.81	77.13
AugMix (Hendrycks et al., 2019)	83.65	75.43	88.26	82.68
M2D (Ours)	**86.08 (85.62–86.64)**	**76.72 (76.15–77.30)**	**92.09 (90.63–93.55)**	**85.98 (84.21–87.75)**
Panel B: RNFL-Specific Single-Layer Evaluation				
M2D (Ours)	**86.10 (85.52–86.68)**	**78.50 (77.85–79.15)**	**92.77 (91.43–94.11)**	**88.21 (86.54–89.88)**

MIoU: mean intersection over union; RNFL: retinal nerve fiber layer; Dice: Dice Similarity Coefficient. Panel A presents metrics averaged across the three primary retinal layers. Panel B evaluates the RNFL, exclusively. For MB-U-Net and the proposed M2D, results are presented as Mean (95% CI) to illustrate performance variance. The pooled RNFL, Dice and MIoU across the entire 1,017-scan target cohort are 87.42% (86.12–88.72) and 80.43% (79.24–81.62), respectively. The metrics reported for the proposed M2D framework represent the optimal model selected via source-domain validation.

Bold values indicate the best performance for each evaluation metric.

**FIGURE 4:**
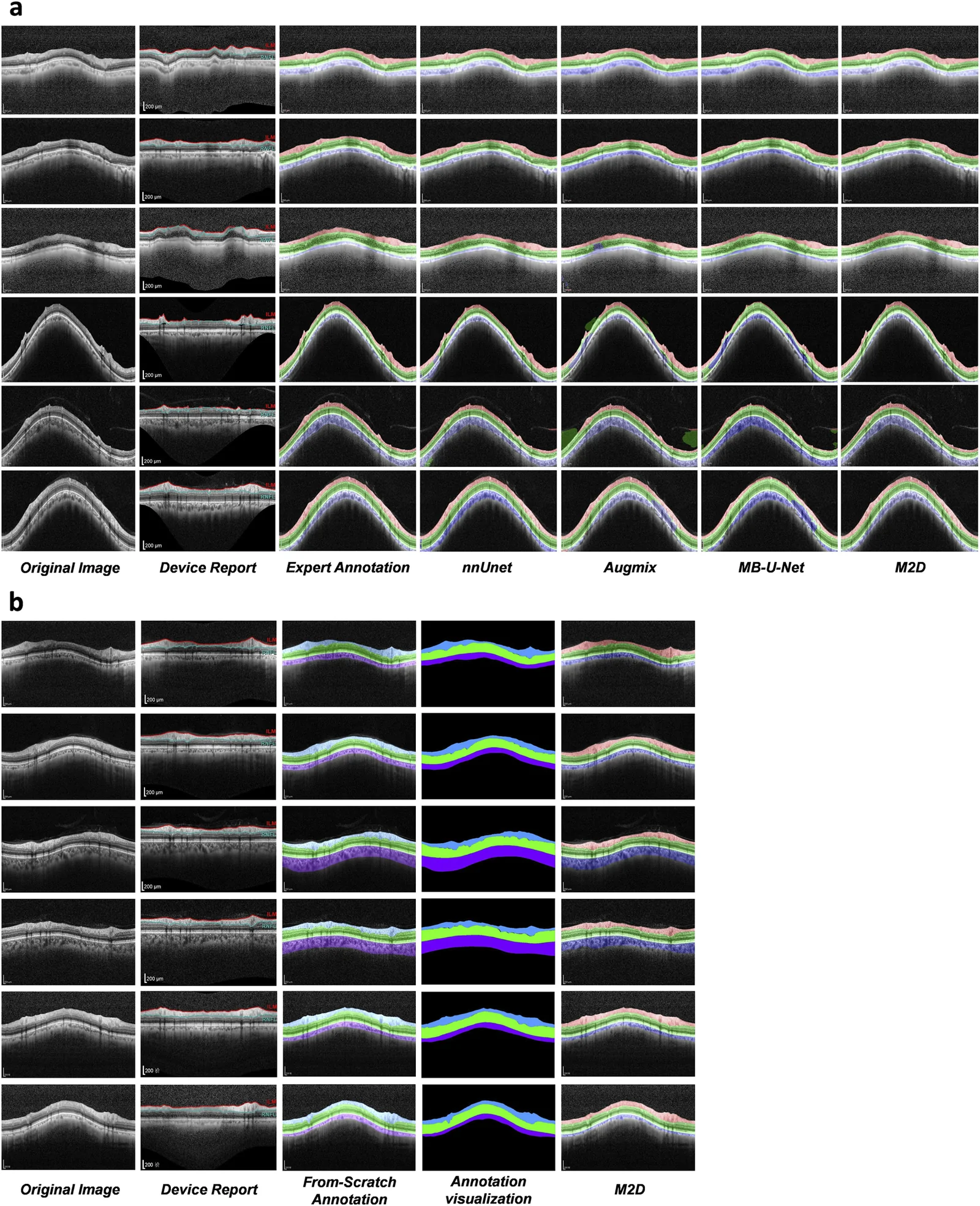
Qualitative comparison and bias validation of three-layer retinal segmentation on target ONH circumpapillary scans. **(a)** M2D outputs are contrasted with the proprietary *Device Report* and the *Expert Annotation* (adjudicated consensus). In contrast to the comparative models, M2D demonstrates boundary adherence consistent with the expert consensus, mitigating the tracking deviations observed in the commercial OCT report. **(b)** Visual concordance between the completely blinded, from-scratch expert annotations and the unedited M2D predictions on a randomly sampled subset. This preliminary comparison provides directional reassurance regarding the annotation process, though a larger independent cohort is required to definitively exclude anchoring bias.

The framework also retained source-domain macular segmentation fidelity, mitigating catastrophic forgetting. Qualitatively (Figure 4), the model demonstrated boundary adherence across pronounced ONH morphological variations. Whereas the MB-U-Net produced topological artifacts in structurally complex regions like steeply sloping neuroretinal rims, the proposed approach preserved anatomical integrity and continuous RNFL delineation.

Ablation experiments (Table 3) verify the functional contributions of the structural and photometric modules. For isolated RNFL segmentation (Table 2, Panel B), the complete framework yielded an overall average Dice of 87.42% (95% CI: 86.12%–88.72%) and an MIoU of 80.43% (95% CI: 79.24%–81.62%) in strict agreement with the commercial reference across the 1,017 target-domain scans, reflecting the algorithmic capacity to approximate standard proprietary outputs across varying clinical cohorts.

**TABLE 3:** Ablation study of the M2D framework components.

Configuration	Heidelberg		TowardPi	
	Dice (%)	MIoU (%)	Dice (%)	MIoU (%)
Baseline (MB-U-Net)	84.04 (83.02–85.11)	74.02 (72.85–75.19)	88.62 (85.81–91.43)	80.66 (77.72–83.60)
+ $A_{s}$	84.99	75.17	88.67	80.84
+ $A_{p}$	85.55	76.41	89.78	81.53
+ $L_{\mathrm{global}}$	84.90	75.27	89.15	81.40
+ $A_{s}$ + $A_{p}$	85.59	75.73	89.96	83.65
+ $L_{\mathrm{global}}$ + $A_{s}$	85.21	76.15	91.78	85.53
+ $L_{\mathrm{global}}$ + $A_{p}$	85.67	76.02	90.50	83.20
Full Framework (M2D)	**86.08 (85.62–86.64)**	**76.72 (76.15–77.30)**	**92.09 (90.63–93.55)**	**85.98 (84.21–87.75)**

Dice: Dice Similarity Coefficient; MIoU: mean intersection over union; CI: confidence interval; $A_{s}$ : Spatial Structure Space (TLDM Deformation); $A_{p}$ : Pixel-level Appearance Space (NURBS Mapping); $L_{\mathrm{global}}$ : Target-Label-Free Global Alignment Loss. 95% CIs are provided for the baseline and full framework to demonstrate the stabilization effect.

Bold values indicate the best performance for each evaluation metric.

### 3.3 Distribution of extracted biomarkers across clinical subgroups

To evaluate the framework’s stability across varying disease severities, algorithmic segmentation outputs were mapped to the confirmed diagnoses of the 422-participant cohort (Table 4). Supported by ground truth from standardized specialist examinations, this phase shifts the focus from pixel-level metrics to assessing the descriptive distribution of AI-derived structural parameters across healthy controls, glaucoma suspects, and advanced cases within the cohort.

**TABLE 4:** Baseline demographics, clinical characteristics, and scan distributions of the stratified study cohort.

Characteristics	Normal Control (n=108)	Cataract Control (n=102)	Suspected Glaucoma (n=110)	Confirmed Glaucoma (n=102)	P -value
Primary Patient Source	Community Outpatient	Outpatient Inpatient	Community Outpatient	Outpatient Referral	–
Key Inclusion Criteria	Normal fundus, IOP ≤21	Slit-lamp confirmed	Screening ${\text{referral}}^{†}$	Tertiary diagnosed	–
Demographics					
Sex (F/M)	58/50	54/48	59/51	57/45	$0.88^{‡}$
Age (Years)	61.5±9.2	65.2±8.7	63.4±9.5	64.8±10.1	$0.14^{§}$
Clinical Parameters¶					
Baseline IOP (mmHg)	14.2±2.9	14.8±3.2	19.6±4.8	22.4±7.5	$<0.001^{§}$
PACD (frac. of CT)	0.94±0.18	0.42±0.15	0.52±0.31	0.58±0.35	$<0.001^{*}$
VCDR	0.34±0.09	0.37±0.11	0.58±0.15	0.78±0.13	$<0.001^{§}$
Scan Cohort (1,017 total)	260	202	280	**275**	–
Heidelberg Spectralis	252	168	235	160	–
TowardPi BMizar	8	34	45	115	–

Data are mean ± SD. SD: standard deviation; IOP: intraocular pressure; PACD: peripheral anterior chamber depth; VCDR: Vertical Cup-to-Disc Ratio; CT: corneal thickness; cpRNFL: circumpapillary Retinal Nerve Fiber Layer. Highly significant differences (P<0.001) in clinical parameters across groups quantitatively confirm the pronounced pathological heterogeneity of the study cohort. ¶Baseline parameters acquired via blinded manual assessment; PACD graded by Van Herick technique.^†^ Referral criteria: IOP >21 mmHg, PACD <0.25 CT, cpRNFL thickness <95 th percentile, or VCDR >0.5 . Statistical Tests:^‡^ Pearson’s $\chi^{2}$ test;^§^ one-way ANOVA;^*^ Kruskal–Wallis H test.

#### 3.3.1 Expert boundary correction and concordance in a mixed-device cohort

To evaluate biomarker extraction fidelity across diverse anatomical presentations, an independent 100-scan mixed-device subset comprising both Heidelberg and TowardPi acquisitions was curated. This cohort encompassed typical morphologies alongside advanced glaucomatous phenotypes. Particular emphasis was placed on cases exhibiting pronounced RNFL attenuation, a pathological state susceptible to boundary-tracking deviations in proprietary algorithms.

To establish a clinical ground truth, an AI-assisted, double-blind annotation paradigm was implemented. Utilizing the unedited M2D segmentation outputs as high-fidelity initial templates, two independent, board-certified specialists performed double-blind manual corrections on the upper and lower boundaries across all three anatomical layers (RNFL, RR, and CC). Subsequently, a senior glaucoma specialist independently reviewed and adjudicated any discrepancies between the primary graders to synthesize the final expert consensus. Prior to consensus adjudication, inter-expert agreement between the two primary graders yielded a CCC of 0.97. Inter-method agreement (M2D versus expert consensus) was quantified using ICC(2,1). It is crucial to emphasize the chronological and functional isolation of this process: these expert annotations were generated exclusively on the target-domain evaluation subset *after* the M2D framework had been fully trained. By utilizing the pre-trained model’s outputs purely as an efficiency tool to expedite ground-truth generation for retrospective evaluation, we ensured these labels were completely isolated from the training and optimization pipelines. This strictly preserves the target-label-free premise of the framework while providing a rigorous baseline for evaluation.

Initially, this AI-assisted annotation paradigm was adopted to mitigate the prohibitive time consumption associated with dense, multi-layer manual delineation from scratch. However, to investigate potential anchoring bias introduced by these M2D initialization templates, an additional subset of 10 independent original OCT circumpapillary scans was randomly selected. A senior ophthalmologist, completely blinded to both the commercial device outputs and the M2D predictions, manually annotated these boundaries entirely from scratch utilizing the open-source LabelMe image annotation tool (version 5.4.1). The statistical comparison between these from-scratch annotations and the unedited M2D predictions demonstrated statistical concordance. We explicitly state these findings as preliminary; while this limited subset is useful as a sensitivity check to provide directional reassurance, it is not sufficient to rule out bias with confidence. Representative visual comparisons are presented in Figure 4b.

Within this rigorously annotated cohort, the proprietary commercial software exhibited a statistically significant deviation from the expert consensus (MAD = 2.8 μm , P=0.04 via GEE, Bias = +1.8 μm ), primarily driven by the overestimation of residual tissue thickness in severely thinned peripapillary regions. Conversely, serving as an initialization baseline, the M2D framework necessitated minimal expert adjustment and achieved statistical concordance in advanced pathology (MAD = 1.8 μm ).

To contextualize these micrometer-scale measurements, a 2.8 μm systematic error is clinically substantial because it exceeds the typical annual rate of glaucomatous RNFL thinning (1–2 μm /year, as further detailed in Section 4.2). To rigorously validate statistical agreement rather than relying on non-significant P -values, we evaluated the mean bias against our predefined strict clinical equivalence margin of ±5 μm . The 95% CI of the mean bias between M2D and the expert consensus was [−0.5 μm,+0.3 μm] . Because this entire 95% CI falls strictly within the ±5 μm margin, statistical equivalence is formally established. By reducing the absolute error to 1.8 μm —well below the hardware’s physical optical limits—the proposed framework demonstrates the sub-pixel precision necessary for consistent cross-platform anatomical measurement. As detailed in Table 5, the performance variance of the proposed framework fell entirely within the observed bounds of expert inter-grader variability.

**TABLE 5:** Quantitative concordance and error analysis of automated cpRNFL delineations against adjudicated expert consensus in a mixed-device clinical subset.

Comparison	MAD (95% CI, μ m)	ICC (95% CI)	CCC	P - ${\text{value}}^{‡}$	Bias ( μ m)	95% LOA
M2D vs. Expert Consensus	1.8 (1.5–2.1)	0.96 (0.93–0.98)	0.96	0.62	−0.1	[−4.3,+4.1]
M2D vs. Blinded From-Scratch	2.1 (1.0–3.2)	0.95 (0.89–0.98)	0.94	0.58	−0.3	[−4.8,+4.2]
Commercial Software vs. Consensus	2.8 (2.4–3.3)	0.93 (0.88–0.96)	0.91	0.04^*^	+1.8	[−4.5,+8.1]
Inter-expert (A vs. B)	1.5 (1.3–1.7)	0.97 (0.94–0.99)	0.97	0.78	−0.05	[−3.6,+3.5]

MAD: mean absolute difference; ICC: intraclass correlation coefficient; CCC: concordance correlation coefficient; LOA: limits of agreement; CI: Confidence Interval.^*^ Indicates statistical significance (P<0.05) . $P^{‡}$ -values were derived via Generalized Estimating Equations (GEE) with cluster-robust standard errors to adjust for intra-subject clustering. Statistical equivalence is evaluated against a predefined clinical margin of ±5 μm .

#### 3.3.2 Anatomical consistency across subgroups

Building upon the established concordance in the mixed-device subset, framework stability was evaluated across the complete 1,017-scan cohort. Proprietary device outputs served as the large-scale baseline comparator, representing standard anatomical delineations in general clinical populations.

Conventional and alternative architectures demonstrated limited adaptability across this heterogeneous dataset. The MB-U-Net systematically underestimated tissue thickness, yielding an overall MAE of 34.8 μ m (P<0.001) . Alternative domain adaptation methods (nnU-Net, AugMix) exhibited elevated variance and statistically significant deviations from the commercial reference ( P=0.03 and P=0.01 , respectively).

In comparison, the M2D structural measurements aligned with the commercial device outputs across all diagnostic subgroups (Overall MAE = 1.5 μ m). The framework’s measurements reflected the progressive structural thinning associated with the disease continuum, documenting average thicknesses from normal controls ( ≈ 110 μ m) to suspected ( ≈ 89 μ m) and confirmed glaucomatous states ( ≈ 69 μ m).

**FIGURE 5:**
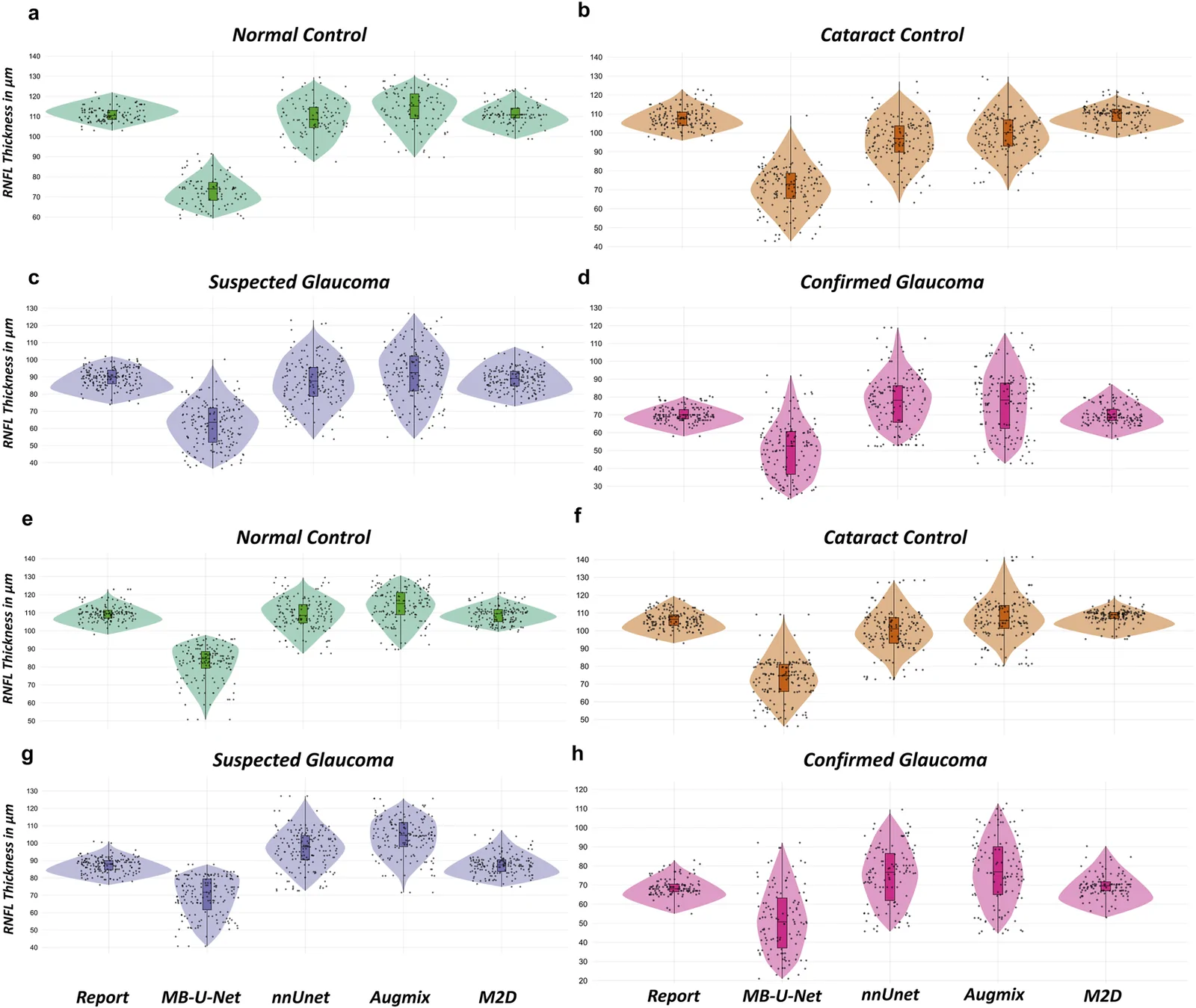
Quantitative evaluation and distribution mapping of cpRNFLT measurements across the full stratified cohort (n=422) . **(a–d)** Correlation analysis and stepwise disease stratification on Heidelberg Spectralis OCT scans, using the proprietary *Report* as the commercial reference. **(e–h)** Corresponding analysis for TowardPi BMizar-400K OCT scans. M2D-derived measurements demonstrate statistical concordance with the *Report* across both hardware platforms, supporting the framework’s exploratory cross-platform consistency for structural anatomical evaluation.

To further investigate cross-platform consistency while acknowledging the inherent clinical confounding within the cohort (Table 6, Panel B), we conducted a stratified analysis utilizing the commercial software as a standardized reference anchor. When adjusting for intra-subject clustering, the M2D framework maintained robust cross-platform consistency between the Heidelberg and TowardPi platforms within all diagnostic subgroups. Crucially, the 95% CI of the overall cross-device difference ( [−0.9,+1.1] μ m) fell strictly within the predefined ±5 μ m clinical equivalence margin, formally establishing cross-platform equivalence without relying on non-significant P -values. Furthermore, the Device × Diagnosis interaction term was not significant $\left(P_{\mathrm{interaction}}=0.12\right)$ , indicating that this measurement stability is maintained regardless of disease severity. The increased absolute deviation from the commercial reference observed in advanced disease stages is consistent with the M2D framework’s demonstrated capacity to compensate for commercial boundary-tracking failures (as independently validated against expert consensus in Table 5), entirely independent of the hardware platform utilized. By profiling these structural biomarkers across disparate hardware platforms (Figure 5; Table 6), the M2D framework demonstrates its utility as a consistent computational methodology for reliable cross-platform structural assessment.

**TABLE 6:** Quantitative comparison of cpRNFLT measurements and stratified evaluation of device consistency across the complete 1,017-scan cohort.

A. Overall cpRNFLT comparison across methods					
Diagnostic Group/Metric	Measured cpRNFLT (Mean ± SD, μ m)				
​	Commercial Reference	MB-U-Net	nnU-Net	AugMix	M2D (Ours)
Normal Control	111.0±4.5	75.2±6.8	110.5±8.2	115.8±7.5	110.8±4.2
Cataract Control	106.8±5.2	71.4±7.5	98.4±10.5	104.2±9.6	108.5±4.8
Suspected Glaucoma	89.5±6.4	61.5±9.8	88.2±12.4	94.6±11.2	89.2±6.1
Confirmed Glaucoma	70.2±5.8	44.8±11.5	73.5±14.8	71.8±13.5	69.5±5.6
Overall MAE (vs. Ref., μ m)	–	34.8 ± 11.4	4.2 ± 3.7	4.8 ± 4.3	1.5 ± 0.8
Overall P -value (vs. Ref.)^§^	–	<0.001 ^*^	0.03^*^	0.01^*^	0.74
Measurement Assessment	Commercial Reference	Severe Underest	High Variance	High Variance	High Concordance

B. Stratified M2D Consistency (vs. Commercial Reference) by Device and Diagnosis					
Metric/Device	Normal	Cataract	Suspected	Confirmed	Overall
MAE ( μ m)					
Heidelberg	0.7±0.2	1.0±0.3	1.6±0.6	2.8±0.8	1.5±0.8
TowardPi	0.9±0.3	1.2±0.4	1.8±0.5	2.6±1.0	1.6±0.8
*P*-value (H vs. T)^||^	0.58	0.64	0.76	0.52	0.61
Dice (%)					
Heidelberg	97.1±1.3	95.8±1.5	92.7±2.1	89.5±2.7	93.8±2.8
TowardPi	96.5±1.5	96.3±1.4	91.9±2.4	90.2±3.1	93.7±2.7
*P*-value (H vs. T)^||^	0.54	0.61	0.58	0.67	0.72

MAE: mean absolute error; cpRNFLT: circumpapillary Retinal Nerve Fiber Layer Thickness; SD: standard deviation; Dice: Dice Similarity Coefficient; Ref.: reference; Underest.: Underestimation. Evaluated on the 1,017-scan cohort (n=422) .

* Indicates statistical significance (P<0.05) . Variance presented for MAE represents the standard deviation across the patient cohort, not algorithmic initialization variance.

^§^ P -values comparing model outputs against the commercial reference were derived via Generalized Estimating Equations (GEE) to adjust for intra-subject clustering.

^||^Stratified Consistency (Panel B): Evaluated using the commercial software as a standardized reference anchor. P -values comparing Heidelberg (H) and TowardPi (T) were computed via GEE with cluster-robust standard errors. To establish formal cross-device equivalence and avoid reliance on non-significant P -values, we calculated the 95% CI for the overall mean absolute difference between platforms. The estimated cross-device difference in MAE was 0.1 μ m (95% CI: [−0.9,+1.1] μ m), utilizing robust standard errors to account for the inherent heteroscedasticity and intra-subject clustering between the distinct demographic cohorts. Because this entire 95% CI falls strictly within our predefined clinical equivalence margin of ±5 μm , cross-platform statistical equivalence is formally established. Furthermore, formal testing for a Device × Diagnosis interaction term in the GEE model yielded no significant effect $\left(P_{\mathrm{interaction}}=0.12\right)$ , confirming that cross-device consistency remains statistically stable across disease stages.

## 4 Discussion

The clinical translation of AI for large-scale ocular evaluation is fundamentally constrained by a dual informatics bottleneck: the prohibitive cost of acquiring expert, pixel-level annotations and the performance degradation induced by domain shifts across heterogeneous OCT platforms (Ting et al., 2019; Devalla et al., 2020). The proposed M2D framework addresses these barriers by providing a target-label-free strategy for cross-anatomical RNFL biomarker extraction, transferring structural priors from readily available macular annotations to unannotated ONH targets.

### 4.1 AI methodology and dual-domain preservation

A central methodological contribution of M2D is its departure from conventional, purely data-driven domain adaptation for clinical image analysis. Rather than relying on target-domain sampling, the AI framework utilizes explicit geometric and photometric modeling. The TLDM abstracts source anatomy into a warp-stable topology, demonstrating that latent structural priors in the macula can be mathematically deformed to resolve ONH annotation scarcity. Concurrently, the NURBS photometric module calibrates disparate device intensity manifolds. By integrating curriculum learning to prevent catastrophic forgetting, the framework achieves dual-domain inference capability, retaining source-domain precision while generalizing to the distinct ONH topology. This biophysically constrained AI approach provides a methodological basis for structural biomarker extraction across varying optical systems.

### 4.2 Biomarker concordance and anatomical fidelity in advanced pathology

Clinical evaluation across the 1,017-scan cohort confirms the technical viability of the M2D framework. Direct application of the MB-U-Net baseline to ONH circumpapillary scans yielded systematic RNFL underestimation ( ≈34 μ m) due to incompatible geometric priors. The proposed AI framework effectively addressed this deficit, aligning with commercial clinical references with a minimal mean absolute error (MAE = 1.5 μ m), which falls well within the predefined clinical equivalence margin of ±5 μ m.

From a clinical perspective, the physiological circumpapillary RNFL is extremely thin (approximately 80–100 μ m in healthy eyes). Because glaucomatous progression typically manifests as a highly gradual structural thinning of merely 1–2 μ m per year (Leung et al., 2012; Miki et al., 2014), reducing an AI’s absolute measurement error by 2–5 μ m is highly relevant for reliable anatomical quantification. This sub-pixel precision assists in distinguishing true structural variations from inter-device noise.

Furthermore, the framework maintained measurement consistency when assessing advanced pathological states. While commercial algorithms perform reliably for healthy anatomies, they exhibited susceptibility to boundary-tracking deviations in cases of pronounced RNFL attenuation, resulting in statistically significant disparities from the adjudicated expert consensus (P=0.04) . As emphasized by our clinical co-authors, this is precisely where the seemingly incremental ∼2.4% improvement in the Dice coefficient carries profound clinical implications. Rather than representing a marginal uniform refinement, this improvement reflects the M2D framework’s capacity to localize the upper and lower RNFL boundaries in regions susceptible to commercial baseline failures, as visually evident in Figure 4a. Grounded by its topology-preserving deformation strategy, M2D consistently delineated these attenuated structural biomarkers, achieving anatomical concordance consistent with the expert consensus. Clinically, this precise boundary extraction is foundational for establishing reliable anatomical baselines in routine clinical practice and mitigating measurement fluctuations driven by software artifacts. It also ensures that structural evaluations across different optical systems are governed by true anatomical morphology rather than algorithmic noise.

However, it is critical to interpret these technical findings within the proper medical context. Glaucoma assessment and diagnosis inherently require a comprehensive, multimodal evaluation that integrates IOP measurements, visual field testing, and detailed optic nerve head morphology assessment. The automated segmentation and thickness extraction of the cpRNFL via OCT represent only one component of this clinical pipeline. Therefore, the proposed M2D framework is not a standalone diagnostic solution, but rather a computational utility designed to support this specific structural assessment within the broader multimodal glaucoma diagnostic workflow.

### 4.3 Limitations and future directions

This study presents several methodological limitations. First, the topological abstraction to three layers ensures biomechanical stability but inherently obscures fine-grained laminar details necessary for evaluating concurrent retinal comorbidities. Second, the 10-scan from-scratch annotation subset serves only as a preliminary sensitivity check; this restricted sample is statistically insufficient to definitively rule out template-induced anchoring bias. Third, extreme geometric augmentation may introduce artifacts in scans characterized by severe signal attenuation ( <15 dB) or complex pathological morphologies (e.g., advanced peripapillary atrophy in high myopia) (Liu et al., 2015; Mansberger et al., 2017).

Fourth, the large-cohort evaluation relies on measurement agreement with commercial OCT software rather than independent expert ground truth. Consequently, the high concordance confirms the algorithmic approximation of commercial baselines, but not absolute accuracy or broad device-agnostic generalization, particularly given the inherent confounding between deployed OCT platforms and clinical case mixes. Future investigations require multi-center datasets with independent expert adjudication and stratified analyses to isolate these specific ocular confounders.

Finally, the current M2D framework remains a retrospective technical prototype operating under a transductive setting. While this design realistically simulates local algorithm calibration upon new device deployment, it precludes immediate out-of-the-box inference on novel distributions. Translating this computational methodology into a clinically actionable tool necessitates exploring inductive domain generalization and conducting prospective validation. Specifically, future deployments must evaluate the system’s structural measurements against direct diagnostic endpoints (e.g., sensitivity, specificity, and ROC/AUC relative to functional visual field status) to establish true clinical utility.

## 5 Conclusion

The Macula2Disc (M2D) framework presents a target-label-free artificial intelligence (AI) strategy to resolve the coupled anatomical and hardware-induced shifts between macular and circumpapillary OCT scans. By integrating topology-preserving structural deformation, device-specific photometric calibration, and unsupervised feature alignment, the framework transfers established macular priors to unannotated optic nerve head targets for automated cpRNFL biomarker extraction.

Testing on clinical data confirms the technical viability of this approach. Specifically, the framework demonstrates structural measurement agreement with commercial software baselines across a heterogeneous 1,017-scan evaluation cohort. Furthermore, when evaluated against adjudicated expert consensus in a limited annotated subset of advanced pathology, M2D demonstrates statistical concordance, mitigating the boundary-tracking deviations observed in commercial algorithms.

Consequently, this study provides a computational methodology for target-label-free segmentation and structural biomarker extraction across varying OCT platforms, establishing a consistent basis for objective cross-device anatomical evaluation.

## Data Availability

The original contributions presented in the study are included in the article/supplementary material, further inquiries can be directed to the corresponding authors.
